# Annealing Effect of Glancing Angle Electron Beam Deposited TiO_2_/In_2_O_3_ Nanowires Array on Surface Wettability

**DOI:** 10.1038/s41598-020-66150-2

**Published:** 2020-06-10

**Authors:** Pheiroijam Pooja, Chinnamuthu P.

**Affiliations:** Department of Electronics and Communication Engineering, National Institute of Technology Nagaland, Chumukedima, Dimapur 797103 India

**Keywords:** Nanoscale materials, Materials science, Structural materials

## Abstract

TiO_2_/In_2_O_3_ nanowire (NW) array are prepared using catalyst free glancing angle deposition technique. The wettability of TiO_2_/In_2_O_3_ NW surface are tuned and controlled by the annealing treatment without altering the surface with additional chemical coating. The phase change, surface roughness, change in static and dynamic contact angles due to the heat treatment are studied. Moreover, the surface properties such as frictional force and work of adhesion are calculated for all the samples. The samples annealed at 600 °C shows nearly superhydrophilic with static water contact angle of 12°, frictional force of 85.00748 µN and work of adhesion of 142.3721 mN/m. The surface of TiO_2_/In_2_O_3_ NW is controlled to attain desired water contact angles and sliding angles, which is paramount for designing practical application in self-cleaning, electronic and biomedical fields.

## Introduction

Surfaces with superhydrophilic wetting nature have been used in numerous applications such as anti-fogging, self-cleaning coatings and tissue reconstruction^1–5^. Controlling the wettability of surface is desirable in various technical applications. In recent years, efforts have been made to improve the hydrophilicity of thin films. There are works on improvement of hydrophilicity using UV illumination of metaloxide films such as TiO_2_, SnO_2_ and ZnO either doping by metal or non-metal^6,7^. However, there are limitations in the application of these metal oxides for use as a reliable superhydrophilic coating and thus need to be checked. For practical application, surface of the film cannot be illuminated with UV light all the time and there is need of long lasting hydrophilicity in absence of UV illumination for anti-fogging surfaces, specifically in outdoor applications. We have reported on designing vertical stacked coaxial TiO_2_-In_2_O_3_ heterostructure nanowire (NW)_,_ which studies on photoinduced hydrophilicity after UV-illumination^8^. There are also reports on hydrophobic to hydrophilic conversion using surface derivatization, chemical grafting, wet chemical reactions and also modifying hydrophobic cross linked high internal phase emulsion polymer (polyHIPE) using styrene^9–11^. Problems associated with these techniques are difficulties in implementation, high cost, polymer degradation possibility and scale up issues for large industrial production.

There is no report on study of tuning the wettability of coaxial TiO_2_-In_2_O_3_ NW from hydrophobic to hydrophilic through simple heat treatment. Moreover, before TiO_2_/In_2_O_3_ NWs are utilized successfully, annealing is necessary to improve the NWs crystallinity. During elevated annealing temperature, the solid-state sintering of TiO_2_ nanostructure subsequently causes structure breakdown. Such results are more noticeable when there is enhanced mass transport and bond breaking during phase transformation from anatase to rutile^12,13^. In case of In_2_O_3_, at elevated annealing temperature the grain stops growing or exhibits amorphous nature^14,15^. Therefore, it is mandatory to elucidate the dependence of thermal stability of desired crystalline phase and nanostructure itself on the annealing temperature, especially for high temperature applications. Detailed study on the structural transformations due to various annealing effects of coaxial TiO_2_-In_2_O_3_ NW structure has not been investigated elsewhere which studies about the phase changes, surface states and surface wettability stability. Further, there is shortage in growth techniques of perpendicularly aligned coaxial NW. Few techniques have been reported for the synthesis of coaxial heterostructure nanowires namely wet chemical synthesis, pulsed laser ablation along with chemical vapor deposition (CVD) and metal organic chemical vapor deposition (MOCVD)^16–18^. In all these methods, growth controlling is difficult and is catalyst assisted. Glancing angle deposition (GLAD) technique is catalytic free and an efficient way for controlling the growth parameters such as morphology, porosity and thickness^19^.

In this paper, 1D TiO_2_-In_2_O_3_ heterostructure NW has been synthesized using GLAD technique. The structure properties for annealed samples are studied. Attempt has been made to compare the phase transition and textural changes under thermal treatment of 1D TiO_2_-In_2_O_3_ NW. The surface properties are also studied to evaluate the annealing effects on samples for understanding its possible applications.

## Results and Discussions

**Figure 1:**
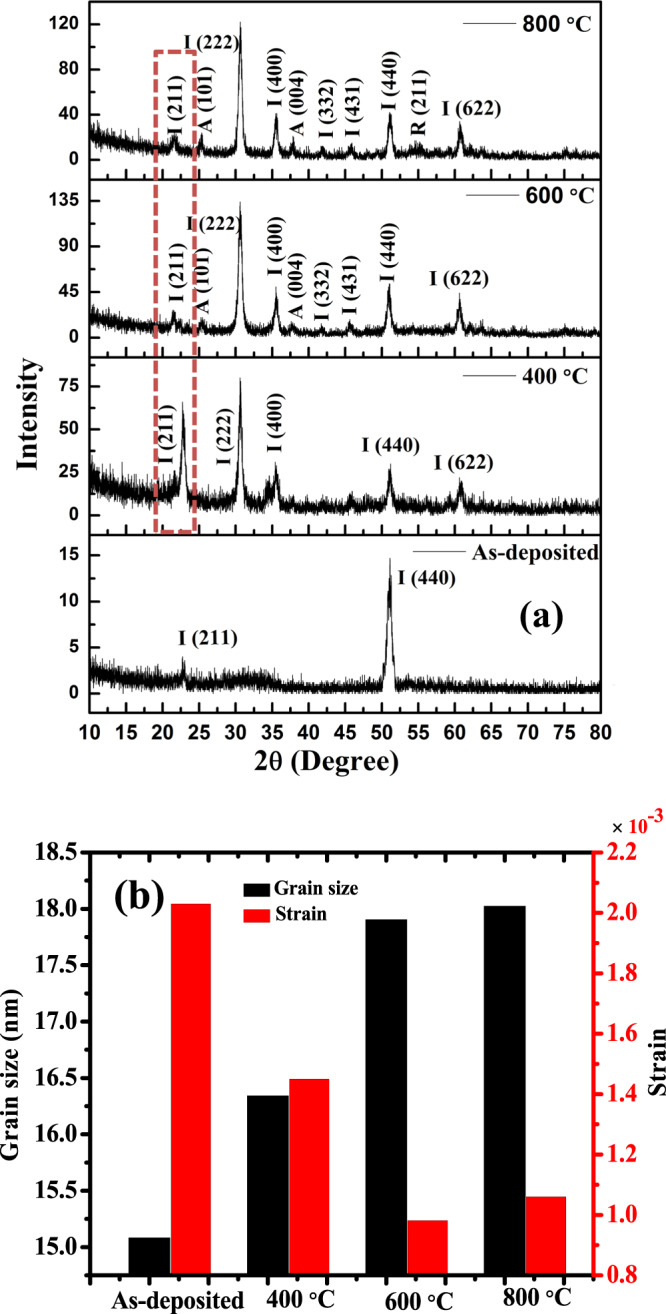
TiO_2_-In_2_O_3_ NWs as-deposited and annealed samples (**a**) XRD and (**b**) size-strain plot.

Figure 1(a) shows the XRD analysis of TiO_2_-In_2_O_3_ NWs deposited on Si (100) substrates as a function of temperature (400–800 °C) and Fig. 1(b) shows the size-strain graph versus as deposited and annealed samples calculated from the Eq. (1) ^20^ given below:

1 $$\beta \,\cos \,\theta =\frac{k\lambda }{D}+4\in \,{\sin }\,\theta $$

Where D stands for crystallite size, λ stands for wavelength of incident light, k is constant (=0.9), θ is bragg angle, β is the full width half maximum of peaks.

The as-deposited TiO_2_-In_2_O_3_ NWs shows two peaks representing planes (211) and (440) of cubic phase for In_2_O_3_ while no peak can be seen for TiO_2_ due to its amorphous nature which agrees with the result reported using e-beam evaporator for deposition^21^. As annealing temperature is increased from 400 °C to 600 °C, In_2_O_3_ exhibited more extra peaks. Increment in the diffraction peak intensities can be observed with the increase in annealing temperature. The peak intensities increment suggest better crystallinity of the samples. There is shift in XRD spectra peak with planes (211) from samples annealed at 600 °C towards lower angle compared to samples annealed at 400 °C. This is due to lattice expansion^22^. TiO_2_ in TiO_2_-In_2_O_3_ NWs is amorphous upto 400 °C and starts to crystallize when annealed at 600 °C and exhibits anatase phase with planes (101) and (004) orientations due to its high stability^23^. Similar result was observed for TiO_2_ annealed above 500 °C^24^. Moreover, the XRD peak intensities of In_2_O_3_ in TiO_2_-In_2_O_3_ NWs increased with the increase in annealing temperature. As the annealing temperature is increased, crystallinity is improved. As a result, strain is reduced till 600 °C. However, for samples annealed at 800 °C, the In_2_O_3_ crystallinity is decreased as peak intensities is decreased compared with samples annealed at 600 °C, where the grain stops growing or exhibits amorphous nature above 600 °C as reported^14,15^. Further, TiO_2_ exhibits mixed anatase-rutile phase diffraction peaks at 800 °C. Yan *et al*. also reported on the appearance of crystallization transformation from anatase to rutile phase above 600 °C^25^. Ma *et al*. reported on collapse of TiO_2_ nanotube arrays structure grown at 800 °C with Ti support in oxygen owing to the mechanical stress and grain growth arising from the ‘feeding effect’^26^. During the phase transformation from anatase to rutile, the TiO_2_ nanowires may distort or disrupt the lattice of TiO_2_. This phase transformation led to break the two Ti-O bonds in the anatase, to rearrange Ti-O in octahedral to form rutile phase^27^. Of all the annealed samples, the sample annealed at 600 °C showed better crystallinity and lesser strain. Lesser strain implies decrease in lattice imperfections^28^.

**Figure 2:**
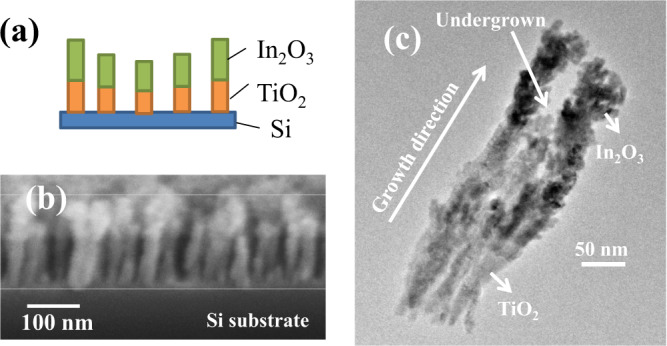
(**a**) Schematics of TiO_2_/In_2_O_3_ NW, (**b**) FESEM cross section image for TiO_2_/In_2_O_3_ NW annealed at 600 °C and (**c**) TEM image for TiO_2_/In_2_O_3_ NW annealed at 600 °C.

Figure 2(a) shows the schematics of vertically aligned TiO_2_/In_2_O_3_ NW array sample and Fig. 2(b) depicts the typical FESEM cross section image of the TiO_2_/In_2_O_3_ NW sample annealed at 600 °C in which the top section is In_2_O_3_ and bottom being TiO_2_. TiO_2_ NWs is deposited beneath the In_2_O_3_ NW to obtain a separated TiO_2_/In_2_O_3_ NWs because In_2_O_3_ has been reported to form interconnected column beyond Zone I structure even under extreme shadowing effect condition due to sufficient diffusion of surface adatoms^29^. In this zone I, the homologous growth temperature parameter (θ = T_S_/T_m_) is less than 0.2 where T_s_ and T_m_ are the substrate temperature and melting point of the deposited material, respectively with little surface diffusion of adatoms take place and the film microstructure and texture are controlled by shadowing^30^. Figure 2a shows that NWs are aligned closely, uniformly and vertically. It can also be seen that some NWs are undergrown due to competitive growth arising from shadowing effect using the GLAD technique. The GLAD technique follows the principle of self-shadowing and vapor fluxes cancellation during azimuthal rotation of substrate to obtain perpendicular aligned growth of NW^31^. Here, with the arrival of vapor fluxes, the taller NW overshadowed the shorter neighboring NWs and as a result, the shorter NWs remain as undergrown NWs.

Figure 2(c) shows the TEM image of annealed TiO_2_/In_2_O_3_ NW sample at 600 °C. In the junction, color contrast can be seen, which confirms the formation of TiO_2_/In_2_O_3_ NW. From the Fig. 2(c), the NW has top and bottom diameter of ~50 nm and ~25 nm respectively. Undergrown region is also seen in the TEM image due to shadowing effect, which plays the main vital role in the formation of NWs.

**Figure 3:**
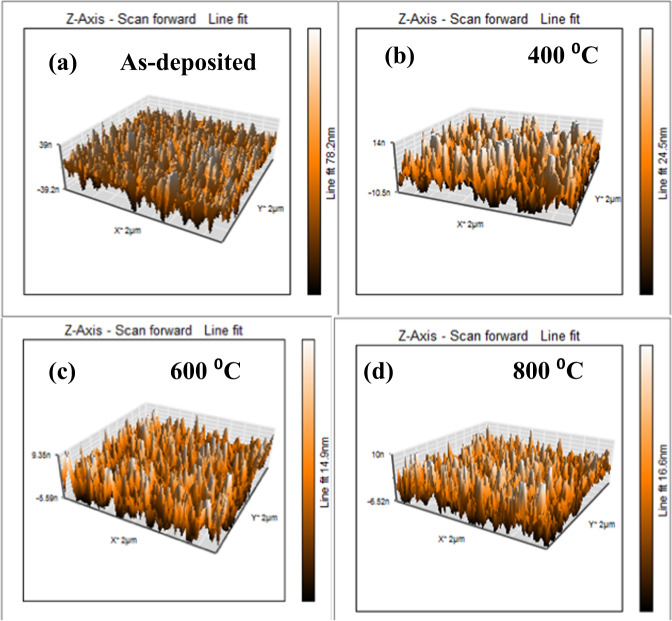
AFM images of TiO_2_-In_2_O_3_ NW (**a**) as-deposited and (**b**–**d**) annealed at 400 °C-800 °C.

Figure 3 shows the AFM images of the as-deposited and annealed (400–800 °C) TiO_2_-In_2_O_3_ NW respectively. The images show recrystallisation due to annealing. With the increase in annealing temperature, the grains initiate to cluster and agglomerate. From the AFM analysis, root mean square (RMS) of the roughness was found to be 10.806 nm (as deposited), 3.867 nm (400 °C), 3.26 nm (600 °C) and 3.54 nm (800 °C). The decrease in the roughness with the increase in annealing temperature is due to the increase in grain size and also the grain agglomeration, which reduces the gaps between the NWs. However, at 800 °C the roughness of TiO_2_-In_2_O_3_ NW increases. This is due to the phase transformation from anatase to rutile; the TiO_2_ NWs may disrupt the lattice of TiO_2_ and thus increases the roughness of the heterostructure^26,27^.

**Figure 4:**
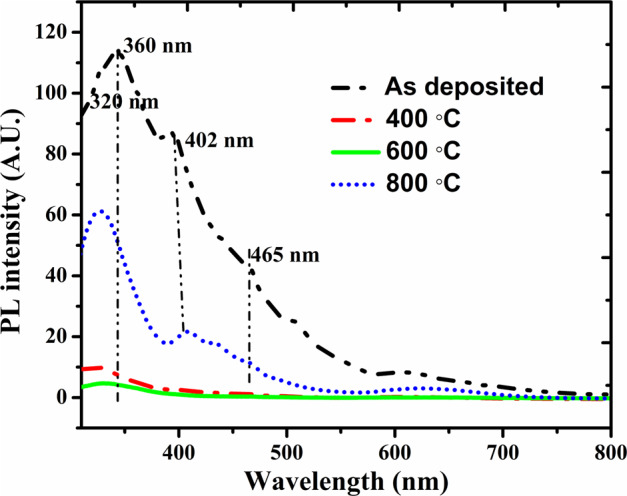
Photoluminescence (PL) analysis of the TiO_2_-In_2_O_3_ NWs as-deposited and annealed samples.

The room temperature PL readings of the as-deposited and annealed samples were recorded by exciting the samples at 250 nm wavelength. Figure 4 highlights the PL emission intensity from the as-deposited and annealed TiO_2_-In_2_O_3_ NW. From Fig. 4 it can be understood that PL intensity decreases as we increase the annealing temperature upto 600 °C and then the emission intensity decrease with further increase in annealing temperature. There is irregularity in the variation of PL intensity with annealing temperature. It has been reported that annealing treatment reduces the number of surface states^32^. On this basis, the PL intensity should shrink upon annealing. However, there can be migration of defects present within the grains onto the grain surface during high temperature annealing treatment. This would raise the amount of surface states, thus enabling the PL emission to enhance to some extent. At 800 °C annealing temperature, oxygen vacancy is created in TiO_2_ and may be the oxygen from In_2_O_3_ might diffuse into TiO_2_ creating oxygen vacancy in In_2_O_3_. As a result, surface state is enhanced and a small shoulder PL emission is introduced at 402 nm (3.08 eV) and 465 nm (2.6 eV) related to oxygen vacancy in In_2_O_3_ and TiO_2_ respectively in TiO_2_-In_2_O_3_ NW samples annealed at 800 °C^33,34^. Moreover, the main-band related PL emission at 340 nm for In_2_O_3_ and 360 nm for TiO_2_ as obtained in our previous work for TiO_2_-In_2_O_3_ NW^35^ is shifted towards shorter wavelength with increase in annealing temperature. This blue-shift cannot be related to quantum confinement^36^ but can be attributed to the widening of energy bandgap with increase in the grain size, which is a function of annealing temperature. Further annealing at 800 °C, the main-bandgap PL emission should be red-shifted due to the formation of lower energy TiO_2_ rutile phase, but the PL emission band is blue-shifted. This may be due to the formation of amorphous Titanium silicate where the Ti ions are diffused out toward the interface during high temperature annealing which cause the blue shift of main band gap emission^24^. It can be concluded that the grain size and annealing treatment can affect the PL emission. Taking these effects into consideration, there is possibility for PL emission to vary irregularly with the annealing treatment as shown in Fig. 4. Among the various annealed samples, samples annealed at 600 °C exhibits reduced PL emission indicating lesser defects.

**Figure 5:**
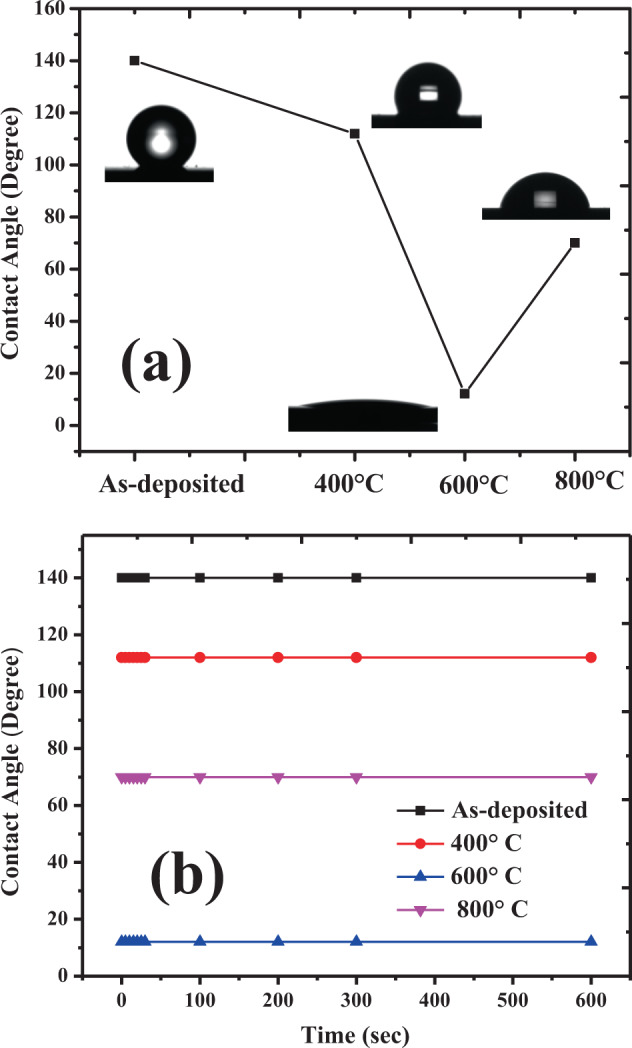
(**a**) Static contact angle of as-deposited TiO_2_-In_2_O_3_ NWs and annealed samples and (**b**) Contact angle versus time plot of TiO_2_/In_2_O_3_ NW as-deposited and annealed samples.

Room temperature static water contact angle (θ_WCA_) measurement was performed on the as-deposited and annealed samples. Figure 5(a) depicts the θ_WCA_ values and water dropping profile of the as-deposited and annealed samples taken during the measurement.

The as-deposited samples exhibits a higher θ_WCA_ of 140° compared to annealed samples. As reported by the author^35^, as-deposited NWs structure are porous due to gap between consecutive NWs. The under-grown NWs arising due to shadowing effect creates more air gaps between the NWs and the air trapped in the gap reduces the fractional coverage at solid-liquid interface which decreases the van der waal forces at the surface. The roughness of AFM is more in as deposited samples. As a result, the θ_WCA_ value of as-deposited sample is higher due to the air trapped between TiO_2_-In_2_O_3_ NWs. Increase in surface roughness enhances the water contact angle which is in consistent with the Cassie and Baxter^37^. Moreover, as seen from the PL analysis, the higher emission from oxygen vacancies in as-deposited TiO_2_-In_2_O_3_ NWs might be one of the reasons for showing higher contact angle. After annealing, the samples θ_WCA_ is reduced compared to as-deposited sample and started showing nearly superhydrophilic nature. This is because after annealing the grain size of the TiO_2_-In_2_O_3_ NWs increases, the NWs starts to cluster and agglomerate which in return reduces the air gap. Since the annealing is carried out in an ambient oxygen environment, this reduces the oxygen vacancies and the oxygen trapped by the surface and hence, θ_WCA_ reduce with the increase in annealing temperature. However, due to the change in phase of TiO_2_ from anatase to rutile at 800 °C, there is lattice distortion which increases the surface roughness of heterostructure and results in trapping of more air, thus enhancing the θ_WCA_ to 70° for TiO_2_-In_2_O_3_ NWs. It can be seen that water contact angle for all the samples is maintained when observed for 10 min as seen in Fig. 5(b).

It is also necessary to characterize the surface wetting behavior using dynamic water contact angle measurement. The advancing contact angle (θ_ACA_) as well as receding contact angle (θ_RCA_) are analyzed through adding and then withdrawing the liquid volume from water droplet^38^. Here, the θ_ACA_ and θ_RCA_ are the angles obtained by liquid expansion and contraction respectively and the resulting contact angle hysteresis (θ_H_) are given in Table 1. The θ_H_ depends on the surface roughness and droplet adhesion to the surface. The value of θ_H_ increased with the increase in annealing temperature due to increase in interaction of surface with water droplet. The as- deposited TiO_2_-In_2_O_3_ NW showed the least difference in the hysteresis. The hysteresis increased with the increase in annealing temperature due to decrease in θ_WCA_. The next parameter, the sliding angle is measured. It is the angle of the droplet at which the droplet starts to slide when the substrate tilts. When the frictional force is low, the water droplet tends to slide effortlessly from the surface. The required maximum frictional force to displace liquid on surface can be found from the following Eq. (2) ^39^:

2 $${F}_{max}=mg\,{\sin }\,\alpha $$

where m is the mass of the water, g is the acceleration due to gravity and α is the sliding angle of the droplet. Another main parameter for wetting of surface is the surface adhesion. It is the attraction of molecules between the surfaces in contact. Work of adhesion (W) between the water droplets and the surfaces of as-deposited and annealed samples are calculated respectively using young-dupre’s formula as given in Eq.(3) ^40^.

3 $$W={\gamma }_{w}(1+\,{\cos }\,\theta )$$

Where, ${\gamma }_{w}$ represents the surface tension of water.

**Table 1:** Dynamic water contact angle measurement.

Samples	Advancing contact angle degree (θ_ACA_)	Receding contact angle degree (θ_RCA_)	Contact angle hysteresis degree (θ_H_)	Sliding angle (α)	Maximum frictional force (F_max_) (µN)	Work of adhesion (mN/m)
As-deposited	179	168	11	20	33.60399	16.54105
400 °C	153	120	33	50	75.21926	44.67327
600 °C	46.6	6	40.6	85	97.66022	142.3721
800 °C	111.3	89	22.3	60	85.00748	96.39072

The as-deposited samples slide the water droplet when the surface was tilted at an angle of 20°. Accordingly, the force that is needed to slide the droplet of water from surface was found to be 33.60399 µN. However, the same droplet was not sliding until the tilt angle of the surface was 85° for samples annealed at 600 °C, which is due to the strong force of 97.66022 µN needed to slide the droplet from the surface. The sliding angle increased with the increase in annealing temperature because of the decrease in water contact angle. The air trapped between the nanowires is reduced due to agglomeration, grain size is increased and surface roughness is minimized. Moreover, hydrophobic surface materials acquire lower work of adhesion. The as-deposited TiO_2_-In_2_O_3_ NW showed lower work of adhesion on comparison with annealed samples as oxygen adsorption tendency is higher at oxygen vacancy sites on the surface in as-deposited TiO_2_-In_2_O_3_ NWs. The as-deposited TiO_2_-In_2_O_3_ NW showed work of adhesion of 16.54105 mN/m, which increased to 142.3721 mN/m for samples annealed at 600 °C. This increase is owing to the increase in contact angle hysteresis. Tuning the oxygen vacancies and structure can change the surface roughness and hence the θ_WCA_. The structure having higher θ_WCA_ can be used in self-cleaning surfaces whereas lower θ_WCA_ can be used in biomedical applications. The surface has been modified for tuning the surface wettability using heat treatment without any chemical surface modification which may be harmful.

### Experimental detail

TiO_2_/In_2_O_3_ coaxial NWs array is deposited on Si<100 > p-type substrate inside e- beam evaporator (BC 300, HHV India) incorporating GLAD technique. Cleaned substrate cut into 1 cm × 1 cm is placed inside the e-beam chamber with base pressure and deposition rate maintained at 6 × 10^−6^ mbar and 0.5 Ås^−1^ respectively. During deposition, the substrate holder is aligned at 85° with respect to the source and also azimuthal rotation of the substrate is maintained at rate of 20 rpm. TiO_2_ NW has been deposited using GLAD technique on Si. Likewise, In_2_O_3_ NW was further evaporated over TiO_2_ NW to obtain TiO_2_/In_2_O_3_ NW. The film thickness was monitored by digital thickness monitor present inside the chamber. To study the annealing effect on the heterostructure NW, the as deposited samples is annealed at various temperatures ranging from 400 °C to 800 °C. Annealing has been executed using muffle furnace, maintaining the same time duration for 1 hr and 6 °C/min heating and cooling ramp rate.

The annealed TiO_2_/In_2_O_3_ coaxial NW morphology was studied using FESEM (SUPRA 55VP, Gemini Column) and transmission electron microscope (TEM). The structural properties of the as-deposited and annealed samples were analyzed using x-ray diffraction (XRD) Cu Kα radiation (RigaKu smart lab guidance). Optical property was studied using F-7000 fluorescence spectrophotometer for photoluminescence (PL) measurement under excitation wavelength of 250 nm. Atomic force microscope (AFM) (Nanosurf, Model: Easyscan 2 AFM) was used to calculate surface roughness of the samples. Contact angle measurement was carried out using sessile drop method with contact angle meter (DMS-401, Kyowa Interface Science CO LTD, Japan).

## Conclusion

Perpendicularly aligned coaxial TiO_2_/In_2_O_3_ NW array are prepared on Si substrate using catalyst free glancing angle deposition technique within electron-beam evaporator. Wettability of TiO_2_/In_2_O_3_ NW surface is tuned using heat treatment in which the surface properties such as phase changes, surface roughness, changes in static and dynamic contact angles are studied. The samples annealed at 600 °C shows nearly superhydrophilic with static water contact angle of 12° and a high sliding angle of 85°. The results indicate that controlling the surface of nanostructure is essential to attain desired water contact angles and sliding angles, which is paramount for designing practical application in self-cleaning and biomedical fields.

## Data Availability

The datasets generated during and/or analyzed during the current study are available from the corresponding author on reasonable request.
